# Mannose-6-phosphate glycan for lysosomal targeting: various applications from enzyme replacement therapy to lysosome-targeting chimeras

**DOI:** 10.1080/19768354.2022.2079719

**Published:** 2022-05-29

**Authors:** Jinho Seo, Doo-Byoung Oh

**Affiliations:** a Environmental Disease Research Center, Korea Research Institute of Bioscience and Biotechnology (KRIBB), Daejeon, Korea; b Department of Biosystems and Bioengineering, KRIBB School of Biotechnology, University of Science and Technology (UST), Daejeon, Korea

**Keywords:** Mannose-6-phosphate, lysosome, glycan, enzyme replacement theraphy and lysosome-targeting chimera

## Abstract

A lysosome, an acidic membrane-bound organelle, contains hydrolytic enzymes to digest macromolecules for recycling. Many lysosomal enzymes (LEs) traffic to the lysosome through the mannose-6-phosphate (M6P)-dependent pathway. Some mannose residues of high-mannose type *N*-glycans on LEs can be phosphorylated in the Golgi apparatus through two-step enzyme reactions. The consequent M6P moiety is recognized by M6P receptors (MPRs) on the *trans*-Golgi network membrane and delivered through the endo-lysosomal pathway. On the other hand, secreted LEs containing M6P glycans can be recaptured by MPRs on the plasma membrane and targeted to the lysosome. Enzyme replacement therapy (ERT) for lysosomal storage diseases exploits this M6P-MPR-dependent endocytosis to deliver recombinant enzymes to lysosomes. This review discusses various engineering and application technologies using M6P’s lysosomal targeting. Glyco-engineering for increasing M6P contents developed ‘Bio-better’ ERT enzymes with enhanced therapeutic efficacy. M6P-decorated peptides, proteins, liposomes, and nanoparticles have been developed for drug delivery and subcellular imaging. A recently developed lysosome-targeting chimera uses an M6P-based bifunctional binder to degrade specific extracellular and membrane proteins. The success and efficiency of M6P-based lysosomal targeting will boost further technological developments with new applications in the biomedical field.

## Introduction

Glycans play essential roles in many critical biological events, such as cell-to-cell interactions, differentiation, development, immunity, and infection, owing to their diverse and unique structures (Reily et al. 2019; Schjoldager et al. 2020). A glycan structural motif for lysosomal targeting was first reported in a pioneering study on fibroblasts derived from a patient with I-cell disease (Hickman and Neufeld 1972); corrective factors (lysosomal enzymes) secreted by most fibroblasts can be taken up for delivery to the lysosome of fibroblasts with a defective lysosomal enzyme (LE) whereas those secreted by I-cell fibroblasts cannot be taken up. The lysosomal targeting motif has been identified as high-mannose glycans containing mannose-6-phosphate (M6P) residues in a study employing M6P addition for competition and alkaline phosphatase treatment for phosphate removal (Kaplan et al. 1977). Further studies have shown that M6P glycans on LEs are recognized by P-type lectins, called M6P receptors (MPRs) (Kornfeld 2018). Most LEs are trafficked from the *trans*-Golgi network (TGN) to the lysosome by MPRs; however, a fraction of LEs is secreted to the extracellular space. Secreted LEs can then be recaptured by MPRs located on the plasma membrane and delivered to lysosomes through MPR-mediated endocytosis (Braulke and Bonifacino 2009; Hasanagic et al. 2015). This process is called the ‘secretion-recapture’ pathway, which forms the basis of enzyme replacement therapy (ERT) for most lysosomal storage diseases (LSDs) (Oh 2015).

LSDs, caused by an LE deficiency, have shared symptoms of abnormal accumulation of undigested macromolecules in the lysosome, leading to multiple tissue/organ failure (Bonam et al. 2019). A prevalent option for treating LSDs is ERT, which exploits intravenous injection of recombinant LEs. Most approved LEs, except those for Gaucher disease, contain M6P glycans for efficient lysosomal targeting (Oh 2015). Because high M6P glycan contents of LEs correlate with enhanced lysosomal targeting efficiency and therapeutic efficacy, many glyco-engineering strategies to increase M6P glycan content have been developed (Oh 2015). The success of lysosomal targeting using M6P glycans in ERT has boosted the development of various applications. M6P-decorated proteins, liposomes, and nanoparticles have been developed for drug delivery and subcellular imaging. The Bertozzi group recently developed an intelligent protein degradation tool called lysosome-targeting chimeras (LYTACs) using an M6P-modified binder for lysosomal degradation of extracellular and membrane proteins (Banik et al. 2020).

This review briefly introduces the M6P glycan pathway for trafficking to lysosomes and focuses on various M6P-based applications.

### M6P glycan biosynthesis and recognition by MPRs

**Figure 1.**
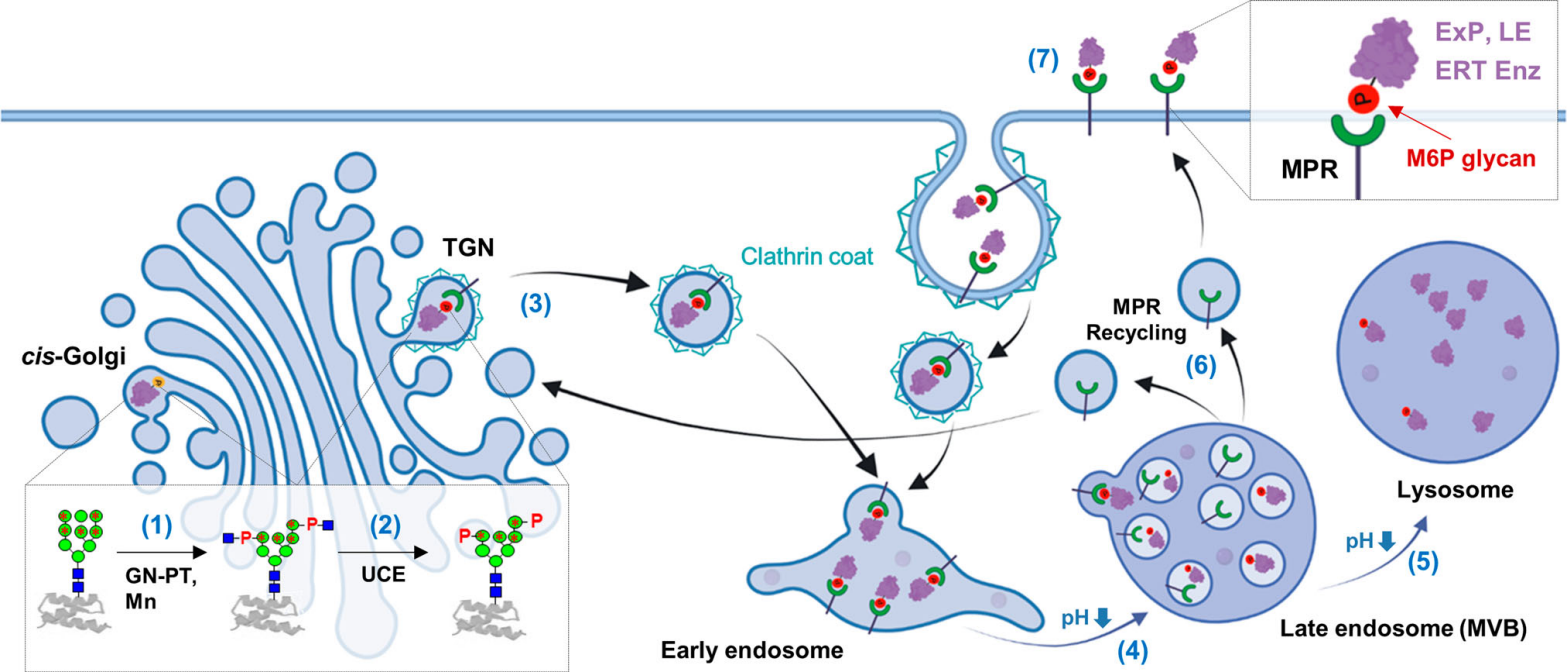
Schematic representation of M6P glycan biosynthesis and its role in lysosomal targeting. In the *cis*-Golgi apparatus, GlcNAc-PT (GN-PT) transfers GlcNAc-1-phosphate to high mannose glycans on LEs, accompanied by mannosidase (Mn) trimming (1). In the TGN, the uncovering enzyme (UCE) removes the outer GlcNAc to generate M6P (2). MPRs at the TGN membrane recognize M6P glycans on LEs, and the LE-MPR complex moves to the early endosome via clathrin-coated vesicles (3). The LE-MPR complex dissociates at the low pH of the late endosome, including the multivesicular body (MVB) (4). The released LEs go to the lysosome (5), whereas the MPRs return to the TGN through the recycling endosome (6). Extracellular proteins (ExP) containing M6P glycans, such as LEs and ERT enzymes, can be taken up by CI-MPR at the plasma membrane through clathrin-mediated endocytosis and delivered to lysosomes via the late endosome (7). Symbols used for glycans are suggested by the Consortium for Functional Glycomics (http://www.functionalglycomics.org/): Green circle, mannose; blue square, GlcNAc; and P, phosphate. This figure was created with BioRender.com. The potential mannose residues for phosphorylation are indicated with an asterisk (*) (Zhu et al. 2005).

From yeast to mammals, all eukaryotes have a common conserved *N*-glycosylation pathway in the ER. In contrast, *N*-glycan processing in the Golgi apparatus is highly divergent across eukaryotic species and generates heterogeneous mixtures (Van Landuyt et al. 2019). Among various *N*-glycan processings, M6P modification is known to occur in the mammalian Golgi apparatus. However, receptors recognizing M6P glycans have also been reported in vertebrates (Castonguay et al. 2012) and invertebrates (Bhamidimarri et al. 2018). Most high-mannose type *N*-glycans (Man_8-9_GlcNAc_2_) produced in the ER are trimmed to Man_5_GlcNAc_2_ glycans in the *cis*-Golgi for further processing into complex glycans. On the other hand, some high-mannose glycans (Man_6-9_GlcNAc_2_) on LEs are recognized in a conformation-dependent manner by GlcNAc-1-phosphotransferase (GlcNAc-PT) (Figure 1) (Bao et al. 1996; Qian et al. 2010). GlcNAc-PT transfers GlcNAc-1-phosphate from UDP-GlcNAc to the C6 hydroxyl groups of specific mannose residues of high-mannose glycans, generating ‘GlcNAc-1-phosphate-^6^O-mannose’ structure. Next, the uncovering enzyme removes the outer GlcNAc, which completes the M6P (phosphate-^6^O-mannose) structure in the TGNs (Rohrer and Kornfeld 2001; Gorelik et al. 2020).

After M6P glycan formation, LEs are recognized by MPRs at the TGN membrane. MPRs are classified as cation-independent (CI)-MPR or cation-dependent (CD)-MPR depending on the requirement of divalent cations for M6P binding (Kornfeld 2018). CI-MPR, which is much larger (300 kDa) than CD-MPR (46 kDa), has 15 homologous domains (each 124–192 amino acids) in the extracellular domain. Domains 3, 5, 9, and 15 (D3, D5, D9, and D15) have the capability of M6P binding with different affinities and specificities, while D11 binds insulin-like growth factor 2 (IGF2); therefore, CI-MPR is also called the IGF2 receptor (Olson et al. 2015; Bochel et al. 2020). The LE-MPR complex is delivered to early endosomes through clathrin-coated vesicles (Figure 1) (Klumperman et al. 1998; Kornfeld 2018). With endosome maturation accompanying a decrease in pH, MPRs dissociate from the LE-MPR complexes at the low pH of the late endosome. The released MPRs are recycled to the TGNs for another round of transport. In contrast, the discharged LEs enter the lysosome through late endosome fusion with the lysosome (Coutinho et al. 2012).

Some fractions of LEs that escape binding to MPRs in the TGN are secreted to the extracellular spaces. These LEs can be recaptured by a small population (∼10%) of CI-MPR existing at the plasma membrane through clathrin-mediated endocytosis (Figure 1) (Braulke and Bonifacino 2009; Oh 2015). ERT enzymes containing M6P glycans exploit this ‘secretion-recapture’ pathway for cellular uptake and lysosomal targeting. In addition to LEs and ERT enzymes, several extracellular and plasma membrane proteins contain M6P glycans; latent TGF-β (Dennis and Rifkin 1991), leukemia inhibitory factor (LIF) (Blanchard et al. 1998; Barnes et al. 2011), CD26 (Ikushima et al. 2000), prorenin (van den Eijnden et al. 2001), proliferin (Lee and Nathans 1988; Blanchard et al. 1998), thyroglobulin (Herzog et al. 1987), granzyme B (Motyka et al. 2000) and EGFR (Todderud and Carpenter 1988). Although these proteins bind to CI-MPR also, their CI-MPR bindings can lead to different results. For example, CI-MPR binding of LIF and EGFR results in their lysosomal degradation. In contrast, granzyme B bearing M6P glycans, after binding to CI-MPR, can enter target cells and induce apoptosis in a perforin-independent manner (Motyka et al. 2000). Furthermore, T cells overexpressing CI-MPR were recently reported to be killed by granzyme B-mediated apoptosis, suggesting a new role for CI-MPR in regulating T cells and adaptive immunity (Ara et al. 2018). CI-MPR can also be employed to activate latent TGF-β and prorenin containing M6P glycans (Dennis and Rifkin 1991; van den Eijnden et al. 2001). Cutting-edge proteomic technologies combining metal affinity chromatography and mass spectrometry have increased the list of nonlysosomal proteins carrying M6P glycans (Caval et al. 2019; Huang et al. 2019; Huang et al. 2021). Notably, CI-MPR is a multifunctional receptor that binds to IGF2, retinoic acid, and plasminogen activation systems, such as urokinase-type plasminogen activator, at each binding site different from M6P binding sites (Brown et al. 2002; Dalle Vedove et al. 2018). In particular, because CI-MPR takes up IGF2 for lysosomal degradation to prevent overgrowth, it was suggested to be a tumor suppressor (Oates et al. 1998; Ghosh et al. 2003).

### M6P glycan is a crucial factor for lysosomal targeting of ERT enzymes

Although more than 50 LSDs have been discovered, only 11 LSDs have been treated with 23 FDA-approved therapeutics, including 15 ERTs, as of May 2019 (Garbade et al. 2020). Except for ERT enzymes carrying mannose glycans for Gaucher disease, the other enzymes require M6P glycans for efficient lysosomal targeting and therapeutic efficacy. M6P glycans on ERT enzymes are recognized by CI-MPR located on the plasma membrane and taken up through clathrin-mediated endocytosis for delivery to the lysosome (Figure 1). Therefore, M6P content often determined the therapeutic efficacy of the ERT enzyme, which stimulated glyco-engineering to develop a ‘Bio-better’ ERT enzyme (Oh 2015).

Fabry disease arises from α-galactosidase (GLA) deficiency, which results in the accumulation of globotriaosylceramide (Gb3) and lysoGb3 in the lysosome. Two approved recombinant human GLAs (rhGLAs), agalsidase alfa and agalsidase beta, are produced from human fibroblasts and Chinese hamster ovary (CHO) cells, respectively (Lee et al. 2003). Both rhGLAs are homodimeric glycoproteins with three *N*-glycosylation sites in each monomer, and two sites are glycosylated with M6P glycans. Agalsidase beta showed better efficacy in Fabry (GLA-knockout) mouse experiments, possibly because of a higher M6P glycan level (Sakuraba et al. 2006). However, a significant difference was not observed in a small clinical study (34 Fabry disease patients) with the same dose treatments (Vedder et al. 2007), later attributed to the marginal difference of M6P contents (agalsidase alfa 2.1 mol/mol and agalsidase beta 2.9 mol/mol) (Togawa et al. 2014).

Pompe disease, caused by acid α-glucosidase (GAA) deficiency, accumulates glycogen in the lysosome, damaging muscle and nerve cells throughout the body. Recombinant human GAA (rhGAA), an algucosidase alfa, was approved by two brand names, Myozyme and Lumizyme, owing to the scale-up problem (Duivelshof et al. 2019). Myozyme is the brand name for the initially approved one, which was produced on a small scale. However, Myozyme was replaced with a new brand name, Lumizyme, because scale-up changed the glycan profile; therefore, the resulting product was considered different from the previous Myozyme. Glycan profiles are considered critical for quality control because glycans play essential roles in hydrophilic structures, clinical efficacy, *in vivo* half-life, and immunogenicity (Duivelshof et al. 2019; Kwon et al. 2021). Therefore, regulatory authorities require that biosimilars have a glycan profile similar to that of the original product. Alglucosidase alfa, produced from CHO cells, was shown to have the lowest M6P content (0.7 mol/mol) in the study comparing the M6P contents of six ERT enzymes (the other enzymes, except the one for Gaucher disease, have 2.1-5.9 M6P mol/mol) (Togawa et al. 2014). Thus, focusing on rhGAA, many glyco-engineering strategies have been developed to increase M6P contents (Oh 2015).

In enzymatic approaches, a two-step *in vitro* reaction using GlcNAc-PT and an uncovering enzyme increased the M6P content of rhGAA bearing high-mannose type *N*-glycan (McVie-Wylie et al. 2008). Although the engineered rhGAA enhanced lysosomal targeting *in vitro*, it did not increase the efficacy of glycogen reduction in Pompe (GAA knockout) mice because the unreacted high-mannose type *N*-glycans provoked clearance by binding mannose-binding lectins and mannose receptors (McVie-Wylie et al. 2008). In contrast, a strategy to overexpress GlcNAc-PT in production cells requires protein engineering of GlcNAc-PT for the detour of its proteolytic activation step (Liu et al. 2017). Engineered GlcNAc-PT (S1-S3) co-expression significantly increased rhGAA’s CI-MPR binding ability (>3-fold) and cellular uptake (2.6-fold), whereas wild-type GlcNAc-PT α/β did not.

Sanofi Genzyme researchers developed a series of chemical conjugation strategies to increase the M6P content. First, they modified M6P glycans isolated from rhGLA and conjugated them to periodate-oxidized sialic acids of rhGAA (Zhu et al. 2004). This conjugation improved lysosomal targeting and enhanced glycogen clearance in Pompe mice. However, this process was inadequate for large-scale production, and the resulting glycans were highly heterogeneous. Second, the M6P glycan optimized for CI-MPR binding (P_2_-Man_6_GlcNAc_2_) was synthesized and conjugated to rhGAA to generate neo-rhGAA (Zhu et al. 2005). In Pompe mouse experiments, compared to rhGAA, neo-rhGAA displayed a comparable glycogen reduction, with an approximately 4- and 8-fold lower dose in the skeletal muscles and heart. Finally, conjugation chemistry was upgraded from a relatively unstable hydrazone bond to a more stable carbonyl-coupled oxime bond, generating oxime-neo-rhGAA (Zhu et al. 2009). This change further improved the CI-MPR binding affinity, which reduced the glycogen level in the skeletal muscle of Pompe mice with 5-fold greater potency than that in unmodified rhGAA. In August 2021, avalglucosidase alfa-ngpt (brand name, Nexviazyme), developed by Sanofi Genzyme, was approved for the treatment of late-onset Pompe disease (https://www.nexviazyme.com/). Avalglucosidase alfa-ngpt containing a 15-fold increased M6P content improved cellular uptake and enhanced glycogen clearance in target tissues.

The glyco-engineered yeast strategy has also been developed to produce ERT enzymes with high M6P content. Although yeasts do not generate M6P glycans, they have mannosyl-phosphorylated mannose residues in the high-mannose type *N*-glycans. This structure (mannose-1-phosphate-^6^O-mannose), resembling ‘GlcNAc-1-phosphate-^6^O-mannose’ in mammals, can be converted to M6P (phosphate-^6^O-mannose) by removing the outer mannose residue (Tiels et al. 2012; Oh 2015). Three engineering steps were required to generate M6P-modified enzymes (Chiba et al. 2002). First, genes producing yeast-specific glycan structures, which can cause immune reactions, were disrupted. Second, the gene coding mannosyl-phosphorylation enzyme was overexpressed to augment ‘mannose-1-phosphate-^6^O-mannose’ structures. Finally, the outer mannose residue was uncapped via an *in vitro* reaction using a glycosidase cocktail solution obtained from a soil bacterium. A recombinant α-mannosidase discovered in *Cellulosimicrobium cellulans* was employed to generate rhGAA containing a high content of M6P glycan (>85%), which showed greatly enhanced efficacy in Pompe mice (Tiels et al. 2012). Our group also developed glyco-engineered yeasts that overexpress mannosyl-phosphorylation enzymes (Gil et al. 2015; Kim et al. 2017). The M6P glycopeptides obtained from the cell wall mannoproteins of glyco-engineered yeast were conjugated to rhGAA, which remarkably increased lysosomal targeting and reduced glycogen levels in Pompe patient fibroblasts (Kang et al. 2018). We also engineered a recombinant mannosyl-phosphorylation enzyme (rMnn14_77-935_) to develop an efficient enzymatic process for M6P glycan generation (Kang et al. 2021).

### Delivery vehicles using M6P

The plasma membrane CI-MPR, a key receptor for lysosomal targeting, is expressed in most tissues, including the kidney, liver, spleen, lung, heart, and muscle, with different expression levels depending on the tissue. On the other hand, it is also reported to be highly overexpressed in some pathological conditions, such as fibrosis, Alzheimer’s disease, and cancers (Dalle Vedove et al. 2018). Therefore, CI-MPR has been targeted for selective anti-cancer drug delivery because of its overexpression in several human cancers, such as melanoma (Laube 2009), pancreatic cancer (Jonson et al. 2001), and prostate cancer (Vaillant et al. 2015). For example, M6P-modified human serum albumin (HSA) has been used as a doxorubicin delivery vehicle for tumors (Prakash et al. 2010). M6P-HSA conjugated with doxorubicin was distributed mainly to tumors and some organs, while free doxorubicin was distributed to all organs. Doxorubicin-M6P-HSA conjugate released doxorubicin at lysosomal pH and inhibited tumor growth, whereas an equal dose of free doxorubicin did not show an anti-tumor effect.

Delivery vehicles using M6P-decorated liposomes have been developed and equipped with a payload for anti-cancer therapy (Dalle Vedove et al. 2018). Liposomes comprising dioleoyl phosphatidylcholine and M6P-functionalized cholesterol were generated and shown to reach lysosomes by observing the fluorescence of the encapsulated calcein (Crucianelli et al. 2014). Furthermore, the same group developed M6P-liposomes loaded with C6-ceramide, which induced lysosomal membrane permeabilization (LMP), accompanied by cathepsin leakage into the cytoplasm and subsequent apoptosis (Minnelli et al. 2018). Ceramide has not been used alone to induce LMP because it forms micelles in an aqueous environment, blocking its cellular uptake and lysosomal targeting. The problem of ceramide delivery to lysosomes was solved using M6P-liposomes. C6-ceramide-M6P-liposomes strongly induced apoptosis in the human breast adenocarcinoma cell line (MCF7) overexpressing CI-MPR, whereas substantial apoptosis was not observed in human dermal fibroblasts expressing a low level of CI-MPR (Minnelli et al. 2018).

The M6P modification has also been used for imaging and theranostics. A multivalent M6P glycopeptide was synthesized and conjugated to a fluorescent probe for active cysteine cathepsins, which was shown to efficiently label cathepsins in the endo-lysosomal compartments of live cells (Hoogendoorn et al. 2014). This report suggests that probes containing the M6P moiety can detect targets located in the endo-lysosomal pathway. For theranostic purposes, the synthesized M6P analog (M6C) was grafted onto the surface of functionalized mesoporous silica nanoparticles (MSNs), covalently incorporating one- or two-photon sensitizers (Vaillant et al. 2015). The resulting M6C-MSNs were used for photodynamic theragnosis to detect and treat prostate cancer cells overexpressing CI-MPR; however, they rarely affected normal prostate tissues expressing a low level of CI-MPR.

### LYTAC using CI-MPR-mediated endocytosis

Targeted protein degradation (TPD) technologies have attracted attention since proteolysis-targeting chimera (PROTAC) showed great promise by degrading ‘undruggable’ target proteins (Verma et al. 2020). PROTACs, which bind both a target protein and an E3 ubiquitin ligase, were designed to induce K48 polyubiquitination of the target protein for proteasome degradation. In addition to PROTACs, various TPD technologies, including molecular glue (Slabicki et al. 2020), autophagy-targeting chimera (Takahashi et al. 2019), and autophagosome-tethering compound (Li et al. 2020), have been developed but most of them can degrade only cytosolic proteins. The Bertozzi group recently developed a new TPD technology for extracellular and membrane protein degradation called LYTAC (Banik et al. 2020).

LYTAC consists of a target protein binder and glycopeptide containing CI-MPR ligands (Banik et al. 2020). Two glycopeptides containing multiple serine-O-mannose-6-phosphonate (M6Pn) or serine-O-M6P residues were synthesized and compared for lysosomal targeting. The M6Pn polypeptides showed better performance than M6P polypeptides of similar length, which might originate from the phosphatase-inert feature of M6Pn. Competition and gene knockdown experiments revealed the essential role of CI-MPR in LYTAC. The crucial role of CI-MPR was highlighted by the observation that surface endosome recycling of CI-MPR was the rate-limiting step in LYTAC. As a proof-of-concept, apolipoprotein E4 (ApoE4), implicated in neurodegenerative diseases, was successfully internalized and degraded in the lysosome by the Poly(M6Pn)-bearing anti-ApoE4 antibody. Furthermore, several membrane proteins (EGFR, CD71, and PD-L1), which are important therapeutic targets, were successfully degraded by LYTAC. The same group developed a second-generation LYTAC employing asialoglycoprotein receptor (ASGPR), a liver-specific lysosome-targeting receptor (Ahn et al. 2021). A triantennary GalNAc ligand for ASGPR binding was conjugated to a binder, such as an antibody or peptide. The resulting GalNAc-LYTAC degraded EGFR and integrins in an ASGPR-dependent manner.

## Conclusion

M6P glycan’s crucial role in lysosomal targeting has been elucidated in studies on lysosome dysfunction. Therefore, many engineering and application technologies have initially focused on ERT to develop ‘Bio-better’ therapeutics for LSDs. The success of M6P-based lysosomal targeting in ERT has boosted the development of other technologies. M6P-decorated proteins, liposomes, and nanoparticles have been developed to deliver drugs selectively to tumors that overexpress CI-MPRs. In particular, the molecules that destroy the membrane integrity of the lysosome showed synergistic anti-cancer effects because they could induce LMP with cathepsin leakage and subsequent apoptosis. The recent development of LYTAC has opened up promising possibilities for generating next-generation therapeutics. Existing TPD technologies, including PROTACs, are limited in that their targets are restricted to cytosolic proteins. LYTAC has been shown to degrade extracellular and membrane proteins, which is also different from target-binding therapeutics, including monoclonal antibodies. The LYTAC technology can be employed to upgrade and repurpose antibodies that have not obtained FDA approval. The strategy using M6P-based lysosomal targeting will provide new applications, especially in the biomedical field.
